# The Role of Calpain-Myosin 9-Rab7b Pathway in Mediating the Expression of Toll-Like Receptor 4 in Platelets: A Novel Mechanism Involved in α-Granules Trafficking

**DOI:** 10.1371/journal.pone.0085833

**Published:** 2014-01-28

**Authors:** Jui-Chi Tsai, Yi-Wen Lin, Chun-Yao Huang, Chih-Yuan Lin, Yi-Ting Tsai, Chun-Min Shih, Chung-Yi Lee, Yung-Hsiang Chen, Chi-Yuan Li, Nen-Chung Chang, Feng-Yen Lin, Chien-Sung Tsai

**Affiliations:** 1 Graduate Institute of Medical Sciences, Tri-Service General Hospital, National Defense Medical Center, Taipei, Taiwan; 2 Division of Cardiovascular Surgery, Tri-Service General Hospital, National Defense Medical Center, Taipei, Taiwan; 3 Departments of Internal Medicine, Taipei Medical University, Taipei, Taiwan; 4 Division of Cardiology and Cardiovascular Research Center, Taipei Medical University Hospital, Taipei, Taiwan; 5 Graduate Institute of Integrated Medicine, China Medical University, Taichung, Taiwan; 6 Graduate Institute of Clinical Medical Sciences, China Medical University, Taichung, Taiwan; 7 Department of Anesthesiology, China Medical University Hospital, Taichung, Taiwan; University of Houston, United States of America

## Abstract

Toll-like receptors (TLRs) plays a critical role in innate immunity. In 2004, Aslam R. and Shiraki R. first determined that murine and human platelets express functional TLRs. Additionally, Andonegui G. demonstrated that platelets express TLR4, which contributes to thrombocytopenia. However, the underlying mechanisms of TLR4 expression by platelets have been rarely explored until now. The aim of this study was to identify the mechanism of TLR4 expression underlying thrombin treatment. The human washed platelets were used in this study. According to flowcytometry and western blot analysis, the surface levels of TLR4 were significantly enhanced in thrombin-activated human platelets and decreased by TMB-8, calpeptin, and U73122, but not Y27632 (a Rho-associated protein kinase ROCK inhibitor) indicating that thrombin-mediated TLR4 expression was modulated by PAR/PLC pathway, calcium and calpain. Co-immunoprecipitation (co-IP) assay demonstrated that the interaction between TLR4 and myosin-9 (a substrate of calpain) was regulated by calpain; cleavage of myosin-9 enhanced TLR4 expression in thrombin treated platelets. Transmission electron microscope data indicated that human platelets used α-granules to control TLR4 expression; the co-IP experiment suggested that myosin-9 did not coordinate with Rab7b to negatively regulate TLR4 trafficking in thrombin treated platelets. In summary, phospholipase Cγ-calpain-myosin 9-Rab7b axis was responsible for the mechanism underlying the regulation of TLR4 containing α-granules trafficking in thrombin-stimulated platelets, which was involved in coagulation.

## Introduction

Platelets are non-nucleated cellular elements that play a critical role in the process of homeostasis and also have roles in innate immunity and inflammation [1]. These anucleate megakaryocyte fragments have the ability to rapidly localize to sites of injury and infection where they release mediators that regulate inflammation and immune progression [2]. Platelets contain stores of cytokines and mediators within their α- and dense-granules that are released upon stimulation. Platelets can also bind to and internalize bacteria and viruses through engulfing, endosome-like vacuoles that fuse with platelet α-granules and allow granular proteins access to the pathogen [3].

Toll-like receptors (TLRs), homologs of the Drosophila protein Toll [4], are pattern recognition receptors that mediate cellular responses to a large array of microbial ligands [5]. Currently, more than 10 different TLRs have been identified; among these TLR4 is a receptor for gram-negative bacteria, LPS, and some viruses. TLR4 is expressed in many different cell types, including dendritic cells, neutrophils, macrophages, epithelial cells, keratinocytes, and endothelial cells [6]–[9]. Recently, both human and murine platelets have been shown to express functional TLR4 [10]–[12], indicating that the TLR4-mediated signaling pathway may contribute to cellular effects in platelets. An additional *in vitro* study demonstrates that LPS accelerates thrombin/collagen-induced aggregation in platelets and that this is mediated by TLR4 expression [13]. Data from *in vivo* assays also show that circulating platelet counts fall precipitously during sepsis and that the degree of thrombocytopenia correlates with the severity of sepsis [10], [14]. Furthermore, platelet counts are decreased under septic conditions due to well-established migration into the lungs and liver [15]. Andonegui *et al.* have demonstrated that TLR4 on platelets is essential for platelet migration into the lungs using adoptive transfer of wild-type or TLR4-deficient platelets into wild-type or LPS-treated mice [5]. It had been reported that platelet TLR4 activates neutrophil extracellular traps to ensnare bacteria in septic situation [16]. Interestingly, extracellular histones promote thrombin generation through the TLR4 on platelets [17]. Thus, platelet TLR4 is proposed to have important roles in platelet function, including platelet adhesion and migration, as well as attraction and destruction. Platelets may also mediate inflammation, immune and aggregation progression via TLR4. However, the underlying mechanisms involved in regulation of TLR4 expression on the surface of platelets are still unclear and remain to be explored. Therefore, we used healthy washed human platelets to examine the expression of TLR4 in thrombin-stimulated platelets in this study and explored its underlying mechanisms *in vitro*.

## Materials and Methods

### Preparation of Washed Human Platelets

Washed platelets were isolated from health male human whole blood by previously described methods [18], [19]. The Institutional Review Board (Taipei Medical University-Joint Institutional Review Board) approved this study, and all volunteers gave written informed consent prior to all procedures. To avoid leukocyte contamination, only the top 75% of the platelet-rich plasma was collected. Briefly, pelleted platelets were washed with HEPES buffer and resuspended in Calcium-free Tyrode's solution. Where indicated, washed platelets (3×10^5^ platelets/µL) were pretreated with inhibitors (in 5% CO_2_ incubation at 28°C 60 minutes) before assaying.

### Reagents

Thrombin, SFLLRN (a PAR1 thrombin receptor-derived hexapeptide), AYPGKF (a PAR4 thrombin receptor-activating peptide) and U73122 (a phospholipase C inhibitor) were purchased from Sigma-Aldrich Chemical Co. (St Louis, MO, USA). Calpeptin (a calpain inhibitor), 3,4,5-trimethoxybenzoic acid 8-(diethylamino)octyl ester (TMB-8; a calcium antagonist), m-3M3FBS (a phospholipase C activator), Y27632 (a Rho-associated kinase inhibitor) and a calpain activity assay kit were supplied by Merck Co. (Frankfurter, Germany). The polyclonal mouse anti-human TLR4 antibody was obtained from Abcam Co. (Cambridge, UK). The monoclonal rabbit anti-human calpain antibody (clone: HPR3319) was obtained from Epitomics Co. (Burlingame, CA, USA). The monoclonal rabbit anti-human tubulin (clone: EP1332Y) and monoclonal mouse anti-human β-actin antibodies (clone: ACTN05/C4) were obtained from GeneTex Co. (Irvine, CA, USA); and the phycoerythrin (PE)-labeled monoclonal mouse anti-human TLR4 antibody (clone: HTA125) was obtained from Biolegend Co. (San Diego, CA, USA).

### Flow Cytometry

Platelets were first fixed with 1% paraformaldehyde for 1 hour at room temperature. Platelets were then stained with phycoerythrin (PE)-labeled monoclonal mouse anti-human TLR4 antibody (clone: HTA125) or a phycoerythrin (PE)-labeled mouse IgG isotype control in the dark. Finally, the platelets were washed with PBS and assayed using a BD FACS Canto II flow cytometer (BD Biosciences, Mountain View, CA, USA) with BD FACS Diva software (Becton Dickinson Immunocytometry Systems, San Jose, CA, USA). The results were collected from 30,000 events.

### Calpain Activity Assay

Calpain activity assays were performed as previously described [20]. This fluorometric assay is based on the detection of cleavage of the calpain substrate Sue-Leu-Leu-Val-Tyr-AMC. Proteolytic hydrolysis of the peptidyl-7-amino bond liberates the highly fluorescent 7-amino-4-methylcoumarin moiety. Quantitation of the 7-amino-4-methylcoumarin (AMC) fluorescence permits the monitoring of enzyme hydrolysis of the peptide-AMC conjugate and can be used to measure enzyme activity.

### Membrane and Total Protein Extraction

Total protein were extracted with lysis buffer (0.5 M NaCl, 50 mM Tris, 1 mM EDTA, 0.05% SDS, 0.5% Triton X-100, and 1 mM phenylmethanesulfonyl fluoride) and membrane proteins were harvested in membrane lysis buffer (20 mM Tris-HCl pH 7.5, 137 mM NaCl, 2 mM EDTA, 1% NP-40, 10% Glycerol, 1 mM PMSF, and 1× protease inhibitor cocktail; Sigma St. Louis, MO, USA) [21]. For membrane fraction, the platelets were followed by three cycles of freeze-thaw then centrifuged to remove the cytoplasmic fraction. The pellets were further washed with lysis buffer to prevent cytosolic protein contamination. Finally, the pellets were resuspended in lysis buffer and designated as the membrane protein fraction. The protein concentration was determined using the Bio-Rad Protein Assay Kit (Bio-Rad, Inc., Hercules, CA, USA) with BSA as the standard.

### Western Blot Analysis

Approximately 50 µg of protein extract was subjected to polyacrylamide gel electrophoresis and then electrophoretically transferred to polyvinylidene difluoride (PVDF) membranes. The membranes were blocked in TBST solution (20 mM Tris-HCl pH 7.5, 138 mM NaCl, and 0.2% NP-40) containing 5% milk powder, and proteins were detected with TLR4 antibody or calpain antibody. Peroxidase conjugated-anti-mouse IgG (Amersham, Arlington Heights, IL, USA) and peroxidase conjugated-anti-rabbit IgG (Amersham, Arlington Heights, IL, USA) were used as secondary antibodies. Peroxidase reactions were carried out and visualized using the chemiluminescence system (Millipore, Bedford, Mass., USA).

### Co-immunoprecipitation (Co-IP) Assay

Total protein was extracted from washed platelets. The protein concentration was determined using the Bio-Rad Protein Assay Kit, and approximately 5 mg of protein extract was precleared with 20 µl of 50% protein A suspension (Bio-Rad, Inc., Hercules, CA, USA) at 4°C for 1 hr. Precleared lysate was then immunoreacted with mouse monoclonal anti-TLR4 antibody (clone: 76B357.1; Abcam, Cambridge, MA, USA) or rabbit anti-myosin heavy chain-9 (myosin-9) antibody (Abcam Co., Cambridge, MA, USA) (2 µg antibody in 1 ml reaction) at 4°C for 16 hrs, and the protein-antibody complexes were then immunoprecipitated by adding 50 µl of 50% protein A sepharose at 4°C for 1.5 hrs. The beads were washed 3 times with extraction buffer. The beads containing immunoprecipitates were then resuspended in 2× SDS-PAGE sample buffer, and the reactions were subjected to western detection and analysis with mouse monoclonal anti-human TLR4 antibody (clone: 76B357.1; Abcam, Cambridge, MA, USA) or rabbit polyclonal anti-myosin-9 antibody (catalog no: GTX13236; GeneTex Co., Irvine, CA, USA).

### Mass Spectrometry

Total protein was extracted from washed platelets and subjected to immunoprecipitation. The immunoprecipitates were eluted with SDS sample buffer, resolved on an SDS-PAGE gel, and stained with Coomassie Brilliant Blue R-250 solution (Sigma, St. Louis, MO, USA). In addition to the TLR4 band, the protein bands common to the anti-TLR4 antibody IP sample but not present in the mouse IgG IP control sample were excised from the gel. The gel pieces were then washed, reduced, alkylated, and digested with trypsin. The tryptic peptides were then analyzed by nano-LC/MS/MS on an LCQ Deca XP Plus ion trap mass spectrometer (Thermo Scientific, San Jose, CA, USA) coupled to an Agilent 1100 HPLC (Agilent Technologies, Inc., San Jose, CA, USA). The MS/MS spectra were searched using Sequest through the Bioworks Browser version 3.3.1 (Thermo Scientific, San Jose, CA, USA) against the NCBI nonredundant protein database.

### Immunogold Staining and Transmission Electron Microscopy

Immuno-gold staining was performed as previously described [22]. In briefly, human platelets were fixed in 4% paraformaldehyde, 0.1% glutaraldehyde, and 0.05% tannic acid in 0.1 M Cacodylate Buffer and embedded in epoxy resin. Prepared 60–90 nm thin sections were detected with rabbit polyclonal anti-TLR4 antibody (catalog no: ab13867; Abcam, Cambridge, MA, USA) and then incubated with 18 nm Colloidal Gold-AffiniPure donkey anti-rabbit IgG secondary antibodies (Jackson ImmunoResearch Laboratories, Inc., West Grove, PA, USA). At the end, the sections were finally coated with saturated Uranyl acetate and Reynold's lead citrate solution. Sections were examined with a Hitachi H7000 transmission electron microscope (TEM) (Hitachi Chemical Co., Ltd. Japan) operating at 75 kV under standard operating conditions.

### Statistical Analysis

The values were expressed as the mean ± SEM. Statistical evaluation was performed using one-way ANOVA followed by the Dunnett test, with a p value<0.05 being considered significant.

## Results

### Thrombin Induces TLR4 Expression on the Surface of Platelets *via* the PAR/PLC Pathway

**Figure 1:**
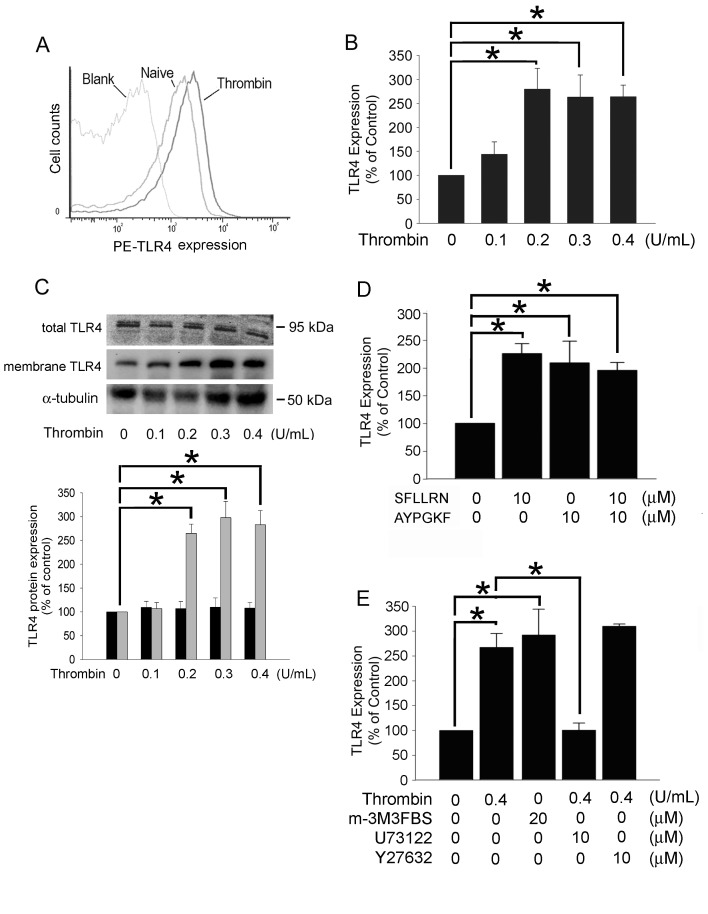
Thrombin induces TLR4 expression on human platelets via the PAR/PLC pathway. (A) Washed human platelets were treated with 0.4 U/mL thrombin at 37°C for 20 min, and the level of TLR4 on the surface of platelets was determined by flow cytometry. (B) Washed human platelets were treated with 0.1–0.4 U/mL thrombin at 37°C for 20 min and analyzed by flow cytometry for the surface level of TLR4 (n = 3). (C) The total and membrane protein fraction were extracted, and the TLR4 levels were further confirmed by western blot analysis and detected with anti-TLR4 antibody. α-tubulin served as the loading control in this assay. The bar graph showed the quantification of western blot analysis using densitometry. (D) Human platelets were directly treated with SFLLRN, AYPGKF, or SFLLRN plus AYPGKF at 37°C for 20 min. The level of surface TLR4 was determined by flow cytometry. (E) Washed human platelets were pretreated with U73122 or Y27632 at 28°C for 60 min followed by 0.4 U/mL thrombin treatments at 37°C for 20 min, and the surface level of TLR4 on the platelets was determined by flow cytometry. The m-3M3FBS was pretreated at 28°C for 60 min, than incubate at at 37°C for 20 min. The data represented the results of 5 independent experiments (mean ± SD; *p<0.05).

A previous study demonstrated that expression of TLR4 on the surface of platelets plays an important role in platelet-related immunity [1]. The mechanisms involved in the regulation of TLR4 expression on the surface of resting or activated platelets are as yet unclear and remain to be studied. Flow cytometry using a phycoerythrin (PE)-labeled mouse anti-human polyclonal TLR4 antibody was performed to determine whether surface expression of TLR4 is increased in activated platelets. As shown in figure 1A, TLR4 fluorescence intensity on the surface of platelets was increased (right shift) in the thrombin-activated group compared with the resting naïve group. Furthermore, stimulation with 0.2, 0.3 or 0.4 U/mL thrombin significantly increased the expression of TLR4 in a dose-dependent manner relative to that of the untreated control group (279.56±74.72%, 263.12±79.16% and 263.75±34.07% of control, respectively) (figure 1B). The stimulation of thrombin did not significantly increase the total TLR4 expression in human platelets. The effects caused by thrombin were further supported by western blot analysis of membrane-bound TLR4 proteins (figure 1C). Previous studies using antagonists or antibodies that block PAR1 and PAR4 activation had indicated that PAR1 mediates human platelet activation at low thrombin concentrations, whereas PAR4 contributes to thrombin-induced platelet activation at high thrombin concentrations [23]–[25]. Thrombin may activate both the PLC and Rho pathways, two major G protein–mediated signaling pathways initiated by Gq and G_13_, respectively, through G protein-coupled receptors [26]. The Flow cytometry showed that SFLLRN, AYPGKF, and SFLLRN plus AYPGKF treatment significantly increased the expression of TLR4 relative to that of the untreated control group (218.79±12.86% of control, 206.89±27.89% of control, and 196.14±10.12% of control, respectively) (figure 1D) suggesting that thrombin acted through PAR1 and PAR4 to activate downstream effects in platelets. Figure 1E showed that m-3M3FBS treatment significantly increased the expression of TLR4 (266.67±37.58% of control). In contrast, prior treatment of cells with U73122 followed by 0.4 U/mL thrombin strongly inhibited thrombin-dependent expression of TLR4 (127.93±10.30% of control) relative to the thrombin-activated group, whereas prior treatment of cells with the Rho-associated protein kinase ROCK inhibitor Y27632 had no effects (279.18±3.35% of control). These results suggested that thrombin might utilize the PLC pathway, but not the Rho pathway, to regulate thrombin-mediated TLR4 expression on the surface of human platelets.

### Thrombin-mediated TLR4 Expression in Platelets is Modulated by Calpain Activity

**Figure 2:**
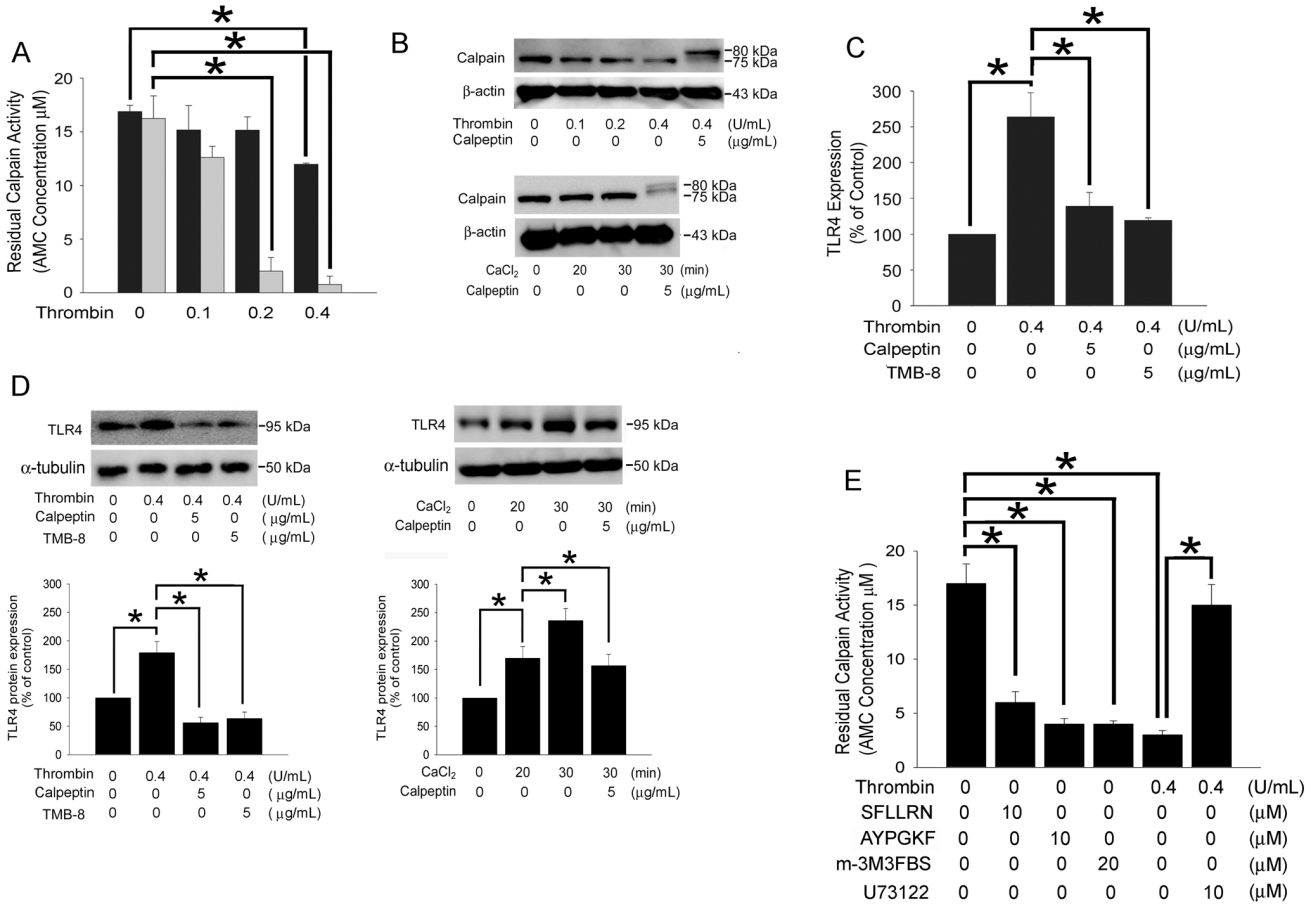
Thrombin-mediated TLR4 expression in human platelets is modulated by calcium and calpain activity. (A) Human platelets were treated with thrombin at 37°C for 1 minute (black) or 20 minutes (gray), and the residual levels of calpain activity were determined. The data represented the results of 5 independent experiments (mean ± SD; *p<0.05). (B) Human platelets were pretreated with calpeptin at 28°C for 60 min followed by thrombin treatment at 37°C for 20 minutes (upper) or 3 mM CaCl_2_ treatment at 37°C for 20–30 minutes (bottom). The total protein was extracted, and the calpain levels were analyzed by western blot and detected with the anti-calpain antibody. β-actin protein served as the loading control. (C) Human platelets were pretreated with calpeptin or TMB-8 at 28°C for 60 min followed by thrombin treatment at 37°C for 20 minutes. The platelet surface TLR4 level was determined by flow cytometry. The data represented the results of 5 independent experiments (mean ± SD; *p<0.05). (D) Human platelets were pretreated with calpeptin or TMB-8 at 28°C for 60 min followed by thrombin treatment at 37°C for 20 minutes (upper) or 3 mM CaCl_2_ treatment at 37°C for 20–30 minutes (bottom). The membrane proteins were extracted, and the TLR4 level was further confirmed by western blot. α-tubulin protein served as the loading control. The bar graph showed the quantification of western blot analysis using densitometry. (E) Human platelets were directly treated with SFLLRN, AYPGKF, or m-3M3FBS or pretreated with U73122 at 28°C for 60 min followed by thrombin treatment at 37°C for 20 min, and the residual levels of calpain activity were determined. The data represented the results of 5 independent experiments (mean ± SD; *p<0.05).

Previous evidence demonstrated that thrombin can induce calcium mobilization in the cytoplasm of platelets and further increases calpain (calcium-dependent, non-lysosomal cysteine proteases) activation [27]. To confirm the roles of calcium and calpain in thrombin-mediated TLR4 expression and to determine whether elevation of intracellular calcium can activate calpain and further increased TLR4 expression in platelets, we treated platelets with calpeptin or TMB-8 followed by thrombin or CaCl_2_ treatment, and the expression levels of membrane-bound TLR4 and cytosolic calpain were assayed. figure 2A showed that intracellular residual calpain activity was significantly decreased in thrombin-stimulated platelets in a dose-dependent manner indicating that the exhausting of calpain activity. Western blot analysis further confirmed that thrombin treatment markedly decreased intracellular residual level of the active form calpain with a size of approximately 75 kDa in a dose-dependent manner in platelets (figure 2B, upper). Prior treatment cells with calpeptin significantly inhibited thrombin or CaCl_2_ induced production of the active form calpain; the calpains present here are in inactive form with a size of approximately 80 kDa (figure 2B, upper lane 5 and bottom lane 4). These results indicated that thrombin might induce calcium mobilization firstly, then increased calpain activation, and finally depleted calpain activity in platelets within a short period of time. To investigate whether calcium mobilization and thrombin-induced calpain activation are involved in thrombin-mediated TLR4 expression on the surface in platelets, we treated platelets with TMB-8 or calpeptin prior to thrombin treatment. The data indicated that calpeptin and TMB-8 significantly decreased thrombin-mediated TLR4 expression, as proven by both flow cytometry and western blot (figure 2C and upper panel of 2D). Additionally, CaCl_2_ treatment markedly increased TLR4 expression relative to the control group; calpeptin pretreatment also significantly inhibited CaCl_2_ induced TLR4 expression (figure 2D, bottom) indicating that calcium mobilization and thrombin-induced calpain activation are indeed involved in this process. Furthermore, SFLLRN, AYPGKF and m-3M3FBS significantly decreased the residual intracellular calpain activity in human platelets. In contrast, prior treatment platelets with U73122 followed by thrombin strongly inhibited residual intracellular calpain activity relative to the thrombin-activated group (figure 2E). This evidences demonstrated that thrombin-mediated expression of TLR4 on the surface of platelets was indeed modulated by both intracellular calcium and calpain activation, which might occur downstream of the PAR1, PAR4 and the PLC pathway.

### The Interaction between TLR4 and Myosin-9, A Novel TLR4-Interacting Protein in Platelet, Is Regulated by Calpain

**Figure 3:**
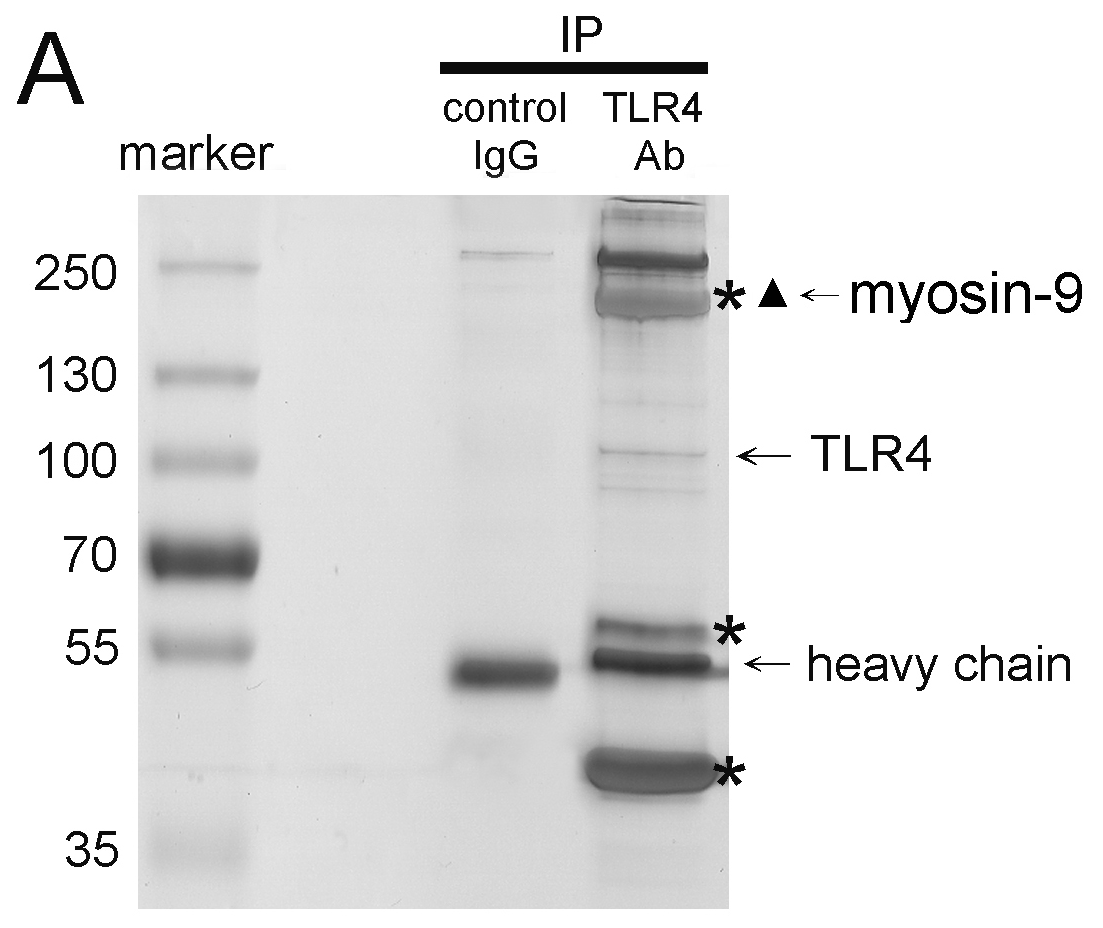
TLR4 interacts with myosin-9. (A) Identification of myosin-9 as a TLR4-interacting protein by co-IP and mass spectrometry. Washed platelet lysates were prepared for IP with mouse IgG- or anti-TLR4-conjugated agarose beads. The precipitated proteins were resolved by SDS-PAGE and revealed by Coomassie Blue staining. The stars indicated the protein bands that were pulled down with the anti-TLR4 antibody but not by mouse IgG. The stars indicated myosin-9 that was identified by nano-LC/MS/MS on an LCQ Deca XP Plus ion trap mass spectrometer.

**Figure 4:**
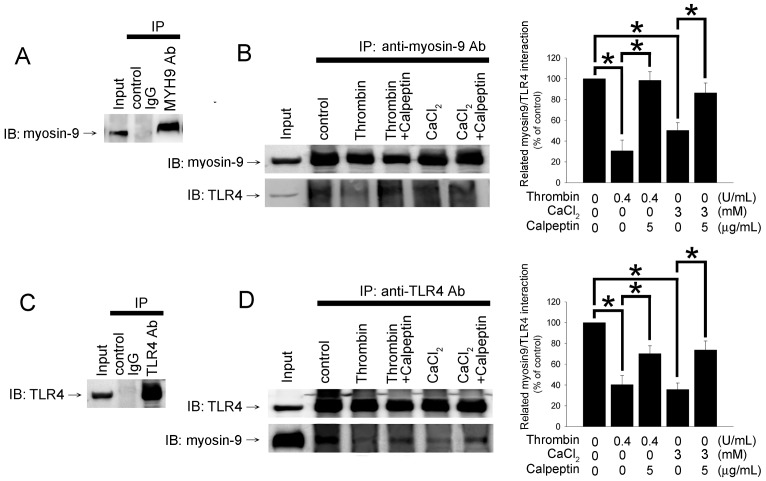
Thrombin-mediated TLR4/myosin-9 interaction in human platelets is modulated by calpain activity. Platelets were treated with 5 µg/mL calpeptin followed by 4 U/mL thrombin for 20 minutes or 3 mM of CaCl2 for 30 minutes. (A and C) The interaction of myosin-9 with TLR4 was analyzed using immunoprecipitation. The pre-immune controls IgG were used to confirm the specificities of the TLR4 and myosin-9 antibodies. (B) The total protein extracted from treated platelets was used for immunoprecipitation with anti-myosin-9 antibody and immunoblotting with anti-TLR4 antibody. Immunoblotting with anti-myosin-9 antibody was used as an IP control. (D) The total protein extracted from treated platelets was used for immunoprecipitation with anti-TLR4 antibody and immunoblotting with anti-myosin-9 antibody. Immunoblotting with anti-TLR4 antibody was used as an IP control. The band density was determined using densitometry and was shown in the graph on the right. The data represented the results of three independent experiments (mean ± SD; *p<0.05 was considered significant).

To explore the molecular mechanisms involved in thrombin-mediated TLR4 expression in human platelets, we identified and characterized TLR4-interacting proteins by using IP and mass spectrometry (figure 3A). Untreated wash platelet lysates were subjected to IP with anti-TLR4 antibody-conjugated agarose beads, and mouse IgG IP was used as a negative control. After being resolved by SDS-PAGE, the precipitated proteins were visualized by Coomassie Blue staining. The protein bands present in the anti-TLR4 antibody IP sample (indicated by stars) but not in the mouse IgG IP control sample were excised for further analysis by mass spectrometry (figure 3A). The mass spectrometry demonstrated that myosin-9 was strongly and consistently present as a ∼220 kDa band and thus chosen for further characterization. IP-mass spectrometry results demonstrated that TLR4 interact with myosin-9 in platelet. Myosin-9 is a part of myosin IIA protein which plays critical role in platelet internal contraction, maintenance of coagulation, differentiation, and cell motility [28]. Additionally, myosin-9 had been demonstrated to participate in cell migration and receptor segregation [29]. We firstly reconfirmed the interaction between TLR4 and myosin-9 by using IP-Western assay. As shown in figure 4B, IP of myosin-9 from platelets with an anti- myosin-9 antibody was performed, and the interaction was analyzed for the presence of TLR4 by western blotting (lane 2). The interaction between TLR4 and myosin-9 was specific because a same result was obtainable when the two antibodies was exchanged (figure 4D). We wander whether the interaction between myosin-9 and TLR4 was involved in the regulation of thrombin-mediated TLR4 expression. Washed platelets were treated with calpeptin and CaCl2 followed by thrombin treatment. The interaction between TLR4 and myosin-9 decreased when the platelets were treated with thrombin and CaCl_2_ (figures 4B and 4D left bottom lane 3 and 5); these phenomena were reversed when platelets were pretreated by calpeptin (figure 4B and 4D left bottom lane 4 and 6). These results demonstrated that myosin-9 might interact with TLR4 in untreated resting platelets; however this interaction would be disrupted when the platelets were under the activation state derived by thrombin or CaCl_2_ treatment. We hence suggested that decreased interaction between myosin-9 and TLR4 was positive associated with thrombin-mediated TLR4 expression; the interaction between myosin-9 and TLR4 was regulated by the calcium-calpain axis in thrombin treated platelets.

### Myosin-9 Is One of the Substrates of Calpain, and Cleavage of Myosin-9 Enhances TLR4 Expression in Thrombin Treated Platelets

**Figure 5:**
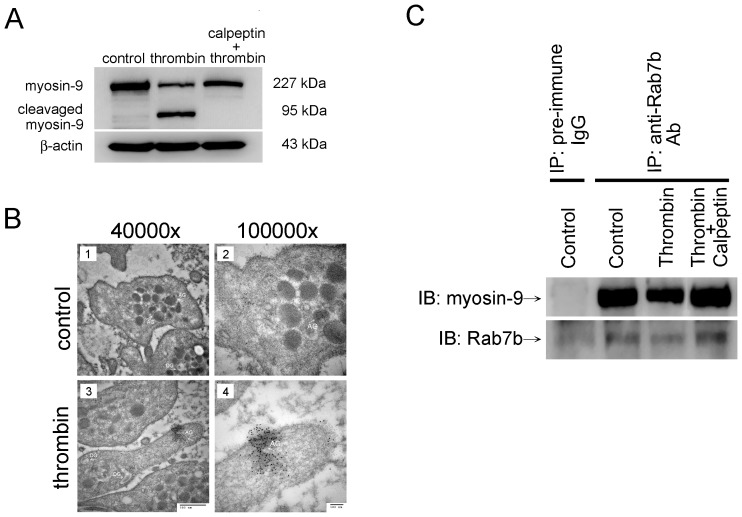
The calpain-myosin-9-Rab7b axis may regulate TLR4 containing α-granules trafficking in thrombin-stimulated platelets. Human platelets were pretreated with or without calpeptin at 28°C for 60 min followed by thrombin treatment at 37°C for 20 minutes. (A) The total protein extracted from platelets was used for western blot analysis and immunoblotting with anti-myosin-9 antibody. The β-actin protein served as the loading control. (B) The morphology of platelets were observed using TEM. Control human washed platelets are showed in B-1 and B-2. The platelet with randomly distribution of α-granules (AG) containing immuno-gold conjugated TLR4 (white arrow). Platelets with thrombin treatment at 37°C for 20 minutes were showed in B-3 and B-4. Original magnification of B-1 and B-3 are 40000×, B-2 and B-4 are 100000×. (C) The total protein extracted from platelets was used for immunoprecipitation with anti-Rab7b antibody and immunoblotting with anti-myosin-9 antibody. A pre-immune control IgG was used to confirm the specificity of the Rab7b antibody. Immunoblotting with anti-Rab7b antibody was used as an IP control.

Several reports demonstrate the importance of calpains in many different platelet activation process including spreading, aggregation, granule secretion, and integrin signaling [30]. Calpain is also known to be responsible for the limited proteolysis of a spectrum of cytoskeleton-associated proteins including spectrin, adducin, talin, filamin, vinculin, and cortactin [30]. Previous evidence had indicated that the activation of calpain leads to the proteolysis of platelet cytoskeleton-associated proteins. This phenomena associated with the change of platelet function [31]. We supposed that myosin-9 might be also one of the substrates for calpain. We had already found that thrombin treatment significantly induced calpain activation and TLR4 expression. Figure 5A showed that thrombin treatment dramatically decreased the level of myosin-9 in platelet relative to the control. Bedsides, thrombin treatment was sufficient to stimulate the degradation of myosin-9 because a cleavaged form myosin-9 with 95 kDa was significantly present in thrombin treated platelets (figure 5A lane 2) but not in other groups. The degradation of myosin-9 was reversed when platelets were pretreated with calpeptin (figure 5A lane 3) indicating that myosin-9 was indeed one of the substrates of calpain. The data indicated that myosin-9, a TLR4-interacting protein in platelet was one of the calpain substrates; cleavage of myosin-9 by calpain and decreased interaction between myosin-9 and TLR4 were positive associated and enhanced thrombin-mediated TLR4 expression in platelets.

### Myosin-9 Did Not Coordinate with Rab7b to Negatively Regulate TLR4 Trafficking in Thrombin Treated Platelets and Enhanced TLR4 Expression in Platelets

In platelets, α-granules containing several growth factors and activation-induced receptors; we predicted α-granules were the major storage compartments for TLR4 in platelets, According to TEM photos, the control platelet had randomly distribution of organelles and the immuno-gold-labeled TLR4 were observed in the α-granules (figure 5B-1 and 2). Interestingly, under thrombin treatment, the α-granules with TLR4 had be moved to the cutting edges of the platelet for release (figure 5B-3 and 4) indicating that platelets used α-granules to delivery TLR4. The figure 5A results let us confidently proposed that calpain mediated myosin-9 degradation and decreased interaction between myosin-9 and TLR4 were positive associated and enhanced TLR4 expression in thrombin-treated platelets. Here we try to figure out the mechanism which was responsible for it. Previous reports had demonstrated that Rab7b protein, a lysosome-associated small GTPase negatively regulates TLR4 expression on macrophage surface by promoting lysosomal degradation of TLR4 [32], [33]. We think that Rab7b guided lysosomal degradation of TLR4 might be also involved in TLR4 expression regulation in platelet. We postulated that myosin-9 might coordinate with Rab7b and negatively regulated the trafficking of TLR4 in resting platelets to keep TLR4 in a basal level state; however this pathway was disrupted in thrombin-stimulated platelets due to the degradation of myosin-9 by calpain activation, and promoted the expression of TLR4 in thrombin-stimulated platelets. We firstly tested whether Rab7b could interact with myosin-9 in resting platelet. IP of Rab7b from platelets was performed by using an anti- Rab7b antibody, and interaction was confirmed by the presence of myosin-9 in western blotting (figure 5C lane 3). We wander whether the interaction between Rab7b and myosin-9 decreased in thrombin-treated platelets. The data showed that interaction between Rab7b and myosin-9 indeed significantly decreased after thrombin treatment (figure 5C lane 4) indicating that thrombin treatment prohibited Rab7b guided TLR4 lysosomal degradation in platelet. Furthermore, the interaction was restored when washed platelets were pretreated with 5 calpeptin followed by thrombin treatment (figure 5C lane 5). Calpain-myosin-9-Rab7b axis was responsible for the mechanism underlying the regulation of TLR4 containing α-granules trafficking in thrombin-stimulated platelets. These results supported that Rab7b coordinated with myosin-9 to negatively regulate TLR4 expression in resting platelet by promoting Rab7b guided lysosomal degradation; however thrombin treatment prohibited Rab7b guided TLR4 lysosomal degradation in platelet result from the cleavage of myosin-9 by calpain activation, and promoted the expression of TLR4 on the surface in thrombin-stimulated platelets.

## Discussion

**Figure 6:**
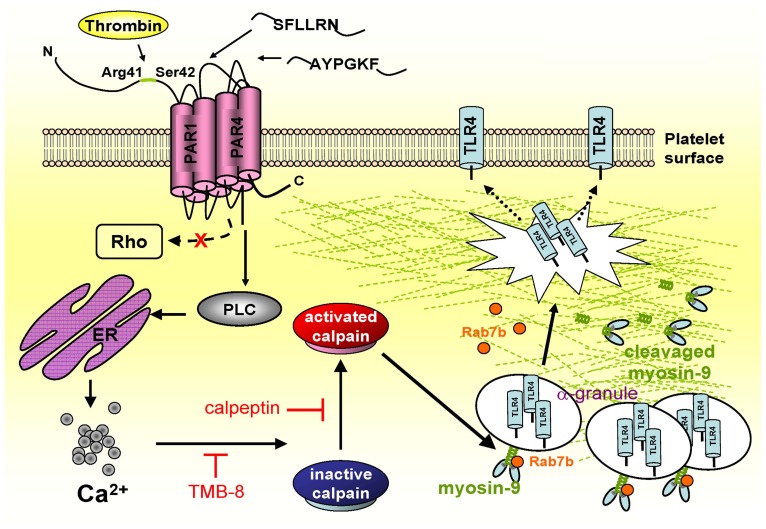
Mechanisms contributing to thrombin-mediated TLR4 expression in platelets. Thrombin may pass through the PAR1 and PAR4 receptors to activate downstream effectors for the PLC pathway but not the Rho pathway. The PLC pathway further activates calpain via calcium mobilization, and cleavages myosin-9, which decreases the interaction between myosin-9 and TLR4. In the other hand, myosin-9 does not coordinate with Rab7b to negatively regulate TLR4 containing α-granules trafficking in thrombin treated platelets, and leads to the increasing of TLR4 performance in thrombin-stimulated human platelets.

Platelet TLR4 expression is associated with sepsis and thrombocytopenia [16]. However, the underlying mechanisms of TLR4 expression by platelets have been rarely explored until now. In figure 6, thrombin-mediated TLR4 expression was modulated by PAR/PLC pathway, calcium and calpain. Additionally, we identified a novel TLR4-interacting protein, myosin-9, which was one of the substrates of calpain; cleavage of myosin-9 enhanced TLR4 expression in thrombin treated platelet. Myosin-9 did not coordinate with Rab7b, a lysosomal-associated small GTPase, to negatively regulate TLR4 trafficking in thrombin treated platelets, and hence enhanced TLR4 expression. Briefly, phospholipase Cγ-calpain-myosin 9-Rab7b axis was responsible for the mechanism underlying the regulation of TLR4 containing α-granules trafficking in thrombin-stimulated platelets, which was involved in coagulation.

TLR4 is expressed on the cell surface of platelets, which regulate immunity and inflammation. In 2004, Aslam R. and Shiraki R. first determined that murine and human platelets express functional TLRs which are potential regulators of innate and adaptive immunity [34]–[36]. In 2005, Andonegui G. demonstrated that platelets TLR4 contribute to the accumulation of platelets in the lungs in response to LPS stimulation [5]. Since then, much work has been devoted to researching the functions and roles of TLR4 in platelets. Platelets express the essential downstream components of the LPS signaling complex, including TLR4/MD2 and MyD88 [13]. Through TLR4-signaling pathway-induced formation of the TLR4/MD2/MYD88 complex, activation of MAP kinase and NF-κB, and production of cGMP, LPS leads to the expression of IL-6, prostaglandin E2, and TNF-α, which is followed by induction of aggregation in platelets [10], [13], [37]. However, TLR4 also causes LPS-incubated platelets to decrease the expression of RANTES, angiogenin and PDGF-AB [38]. Additionally, during a trauma situation or severe sepsis, platelets may respond to LPS through TLR4 to activate neutrophil extracellular traps to ensnare bacteria [16], [39]. In 2007, Dr. John W. Semple *et al.* demonstrated that the LPS from bacteria together with antiplatelet antibodies bound to platelets significantly enhances Fc-mediated platelet phagocytosis by mononuclear phagocytes, which may affect platelet destruction *in vivo*. This mechanism is relevant to the destruction of platelet in autoimmune thrombocytopenia patients with Gram-negative bacteria infections [35]. Although evidence has demonstrated that TLR4 expression on platelets is associated with inflammatory responses, few studies have explored the link between TLR4 on platelets and homeostasis and coagulation. Previously, Kozawa *et al.* explored ADP-induced HSP27 phosphorylation in platelets and found that it was sufficient for granule secretion [40]. Additionally, evidence has been presented in 2012 that the phosphorylation of HSP27 is correlated with ADP-induced platelet aggregation [41]. Although it remains unknown how TLR4 affects platelet aggregation, we speculate that platelets may release HSP27 to regulate the phenomenon through the TLR4 pathway. In fact, we are focusing future work on the mechanisms of TLR4-induced platelet aggregation.

Platelets contain alpha granules, dense granules, and lysosomes that activated platelets may secrete into the blood. This granule secretion is mainly regulated by the cytoskeletal signaling pathway; accordingly, the cytoskeleton may increase their hydrolysis and reorganization. There are many types of proteases with potential roles in the assembly and stability of cytoskeletal signaling complexes. This signaling may result in the association of the receptor with the cytoskeleton, phosphorylation of proteins, an increase in intracellular calcium level, and activation of calpain [42]. The calpain play important roles in physiological activation of platelets. Activation of calpain leads to regulation of SERCA-2 (an intracellular pump possesses Ca^2+^ signaling in platelets), SNAREs (proteins involve in degranulation of platelets), and cytoskeleton proteins (proteins responsible for aggregation, clotting, and shape change in platelets) [30]. Yuan *et al.* reported potentially important roles for calpain in regulating cytoskeletal signaling in vWf-stimulated platelets [31]. Additionally, calpain also cleaves the cytoplasmic domain of the integrin β3 subunit which regulates the activation of the extracellular fibrinogen-binding function of αIIbβ3 and aggregation of platelets [43]. In this study, we firstly demonstrated that calpain had the ability to cleavage myosin-9, a TLR4 interacting protein, and lead to that myosin-9 could not coordinate with Rab7b to negatively regulate TLR4 trafficking in thrombin treated platelets, and hence enhanced TLR4 expression. Although platelets express functional TLR4 mediating neutrophil-dependent pulmonary sequestration [5], whether calpain contributes to thrombocytopenia in response to LPS is still unknown. Similarly, the experiments presented in this study demonstrate that platelet calpain activity is related to TLR4 expression after stimulation of thrombin; whether calpain activity correlates with TLR4-dependent platelet aggregation remains to be elucidated.

The *MYH9* gene, encoding myosin-9 (known as nonmuscle myosin heavy chain IIA), is the only myosin heavy-chain isoform present in platelets [44]. Myosin-9 plays potential roles in maintenance of cell shape and motility, secretion of cytokinesis and growth factors, and formation of platelets from megakaryocytes [45], [46]. In 2009, Tal *et al.* also showed that myosin-9 (myosin IIA) is essential for T cell antigen receptor signaling and immunological synapse stability [28]. Calpain was rapidly activated after calcium ionophore stimulation, and its activity was shown to be essential for the proteolysis of myosin [47]. Myosin-9 consists of four light chains and two heavy chains. The head region of the heavy chains is the major part that interacts with actin to maintain cell movement and shape. Recently more than 300 proteins can be released from activated platelets. A large number of these proteins are carried in α-granules, which are released in response to stimulation in platelets [48]. There are many complex mechanisms regulating the exocytosis of α-granules. Previous evidences explored that Rab4 and Rab6 are essential regulators of the vesicle trafficking and α-granule secretion [49], [50]. Rab6, Rab8, myosin II, and myosin VI had been implicated in trans-Golgi network (TGN)-to-membrane antegrade transport [51]. In contrast, Rab7b is required factor for retrograde transport from endosomes to TGN which mediating lysosomal function [52]. Rab7b is a negatively modulator of TLR4 and TLR9 signaling by leading the translocation of TLRs into lysosomes for degradation in mouse macrophage [33], [53]. Myosin-9 also interacts with the tail regions of other myosin proteins through its long tail region. Although we have explored that myosin-9 may interact with Rab7b and this interaction may regulate the expression of TLR4 in platelets treated with thrombin, the detailed underlying mechanisms and the binding region on myosin-9 remain to be studied in the future. Our mass spectrometry results also showed that in addition to myosin-9, filamin A strongly interacted with TLR4. Human filamin A in is a widely expressed protein encoded by the *FLNA* gene [54], and it regulates actin reorganization by interacting with integrins, second messengers, or transmembrane receptors. Recent evidence demonstrated that thrombocytopenia may result from mutations in filamin A [55]. In the future, it will be important to explore the impact of filamin A on TLR4 expression during the process of platelet activation.

Our results highlight the important roles of phospholipase C_γ_ and calcium-dependent calpain activation in TLR4 expression in thrombin-stimulated platelets. We also explored novel roles for myosin-9 and Rab7b in platelet TLR4 expression through its interaction with TLR4. These primary data indicate a likely mechanism for thrombin-stimulated platelet aggregation and TLR4 expression and also provides a basis for further investigation of TLR4 modulation as a therapeutic strategy for coagulopathy and platelet disorders.
