# Correction: The Role of Calpain-Myosin 9-Rab7b Pathway in Mediating the Expression of Toll-Like Receptor 4 in Platelets: A Novel Mechanism Involved in α-Granules Trafficking

**DOI:** 10.1371/journal.pone.0111995

**Published:** 2014-10-24

**Authors:** The PLOS ONE Staff

There is an error in affiliation 1 for author Jui-Chi Tsai. Affiliation 1 should be: Graduate Institute of Medical Sciences, National Defense Medical Center, Taipei, Taiwan.
